# Effects of feeding hyperlipidemia rats with symbiotic oat‐based frozen yogurt on serum triglycerides and cholesterol

**DOI:** 10.1002/fsn3.949

**Published:** 2019-02-06

**Authors:** Ruishu Yang, Cuina Wang, Haiqing Ye, Feng Gao, Jianjun Cheng, Tiehua Zhang, Mingruo Guo

**Affiliations:** ^1^ College of Food Science Northeast Agricultural University Harbin China; ^2^ Department of Food Science College of Food Science and Engineering Jilin University Changchun China; ^3^ Department of Nutrition and Food Sciences College of Agriculture and Life Sciences The University of Vermont Burlington Vermont; ^4^ Key Lab of Dairy Science Ministry of Education Harbin China

**Keywords:** hyperlipidemia rats, oat‐based frozen yogurt, serum cholesterol, serum lipids, serum triglycerides

## Abstract

Ice cream is one of the most popular dairy foods in the world. But it has long been recognized as a high‐calorie food and may not be suitable for certain segments of the population. Oat‐based food products are considered as functional foods due to its rich β‐glucan. A symbiotic oat‐based frozen yogurt (SOFY) containing fermented oat, probiotics, and inulin (prebiotic) was developed, and its effects on serum lipids in rats were evaluated. The new product had 4.10% protein, 8.50% fat, and 2.10% fiber, respectively. *Bifidobacterium* remained 10^6^ CFU/ml up to 8 weeks. Triglycerides levels of the rats fed with 30% or 35% SOFY were significantly lower than that of the control group (*p* < 0.05). Serum total cholesterol and low‐density lipoprotein levels of experimental rat groups decreased with increased SOFY, and the difference was very significant between group with 35% SOFY and control groups (*p* < 0.01). There was no significant difference in high‐density lipoprotein between each two groups (*p* > 0.05). Hepatic cell cords of rats after administration of SOFY showed orderly manner and normal hepatocyte morphology compared with those of rats in control group. Results indicated that consumption of oat‐based frozen yogurt could lower serum lipids levels in rats.

## 1 INTRODUCTION

Cardiovascular disease (CVD) continues to be an important public health challenge (Anavekar et al., 2004). Dyslipidemia is one of the most potent accepted risk factors for CVD (Kratz, Cullen, & Wahrburg, 2002). Dietary factors, such as consumption of a diet high in cereal fiber, fruits, and vegetables, have been shown to be associated with reduced risk of dyslipidemia and CVD itself (Rimm et al., 1996).

Oat (Avena Sativa L.) is a minor grain crop and a good source of protein, fat, minerals, B‐complex vitamins, and especially heart‐healthy soluble fiber β‐glucan for both human beings and animals (Zhang, Hu, Zhen, Xu, & Fan, 2012). As early as 1963, Biörklund, van Rees, Mensink, and önning (2005) observed that oats lowered serum total cholesterol concentration in humans. Since then, many studies have confirmed this effect and suggested that it is related to the soluble fibers (β‐glucan) in oats (Tiwari & Cummins, 2011). On this basis, oats are claimed to be beneficial for prevention of CVD. However, consumption of oat‐based products is generally low due to lack of acceptable food products formulated with oats.

Probiotics such as *Bifidobacterium* and *Lactobacillus acidophilus* have various benefits including increased resistance to infectious diseases, regulation of hypertension, and decrease in serum cholesterol. Prebiotics are nondigestible food ingredients that selectively stimulate the growth of beneficial microorganisms in the gastrointestinal tract (Ooi, 2010). The interaction between probiotics and prebiotics has been studied for years. Under the condition of their coexistence, activity of probiotics can be enhanced, including cholesterol‐lowering effect on humans (Xie et al., 2011). Soy protein isolate is a high protein (>90%) of soy product. Several studies have shown that soy protein may have a plasma cholesterol‐lowering effect (Sirtori et al., 1995).

Ice cream is one of the most popular dairy foods in the world. But it has long been recognized as a high‐energy food and may not be suitable for certain segments of the population, such as patients with CVD. With the aim to produce a new functional ice cream product, oat was first fermented as the base and then added with soy protein isolate, probiotics (*Bifidobacterium* and *Lactobacillus acidophilus*), and inulin (prebiotic). The novel product was named as symbiotic oat‐based frozen yogurt (SOFY) in this study. The product was expected to have effect of lowering blood lipids. To test this hypothesis, effects of SOFY on serum triacylglycerols (TG), total cholesterol (TC), high‐density lipoprotein cholesterol (HDL‐c), and low‐density lipoprotein cholesterol (LDL‐c) levels in rats were investigated. Pathological changes in liver tissue were also examined. In this study, Wistar rats were used to conduct the experiment, which was the most used effective alternative to mimic the metabolic disease such as cardiovascular diseases.

## 2 MATERIALS AND METHODS

### 2.1 Materials and reagents

Oatmeal was purchased from Guangxi Hezhou Ximai Biological Food Co., Ltd. (Hezhou, China). Probiotics (*Bifidobacterium* and *Lactobacillus acidophilus*) were purchased from Chr. Hansen Co., Ltd. (Milwaukee, WI, USA). Inulin was obtained from Gansu Likang Nutrition Food Co., Ltd. (Dingxi, China). Soy protein isolate and other materials used in this study were purchased from Henan Jincheng Biological Technology Co., Ltd. (Zhengzhou, China). All materials mentioned here were of food grade. All reagents were of analytical grade and purchased from Beijing North Fine Chemicals Co., Ltd. (Beijing, China).

### 2.2 Instruments and equipment

A IKAC‐MAG HS7 magnetic stirring apparatus and a RW20 digital overhead mechanical stirrer were from IKA Works GmbH & Co., Ltd. (Germany); a COMBI M42 ITS ice cream maker was manufactured by Tangning Machine Co., Ltd. (Nanjing, China); a ZG‐2 vacuum freeze drier was made by Hangzhou Creative Vacuum Freeze Drying Equipment Factory (Hangzhou, China); a FW200 high‐speed universal crusher was from Zhongxing Weiye Instrument Co., Ltd. (Beijing, China); and a CX31 microscope was produced by Olympus China Co., Ltd. (Beijing, China).

### 2.3 Preparation of diets

**Figure 1:**
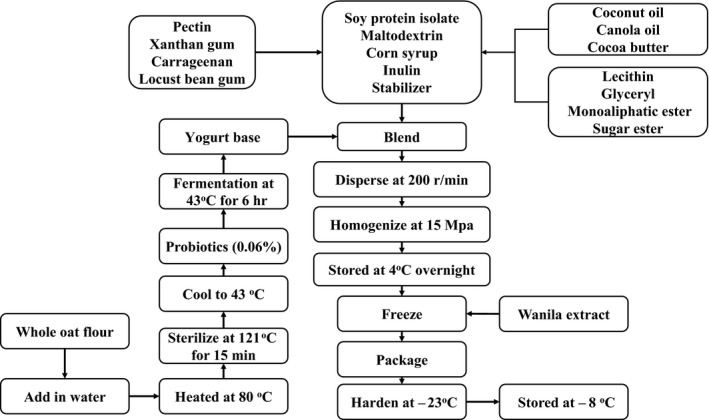
Flowchart of preparation of the symbiotic oat‐based frozen yogurt

Formulation of SOFY (calculated as w/w): oats, 10%; soy protein isolate, 3%; inulin (prebiotic), 0.60%; white sugar, 7.20%; corn starch syrup, 5.60%; maltodextrin, 1.68%; colza oil, 4.43%; coconut oil, 1.05%; cocoa butter, 2.22%; and others, 1.09%. The flowchart for preparation of the oat‐based frozen yogurt is shown in Figure 1. The new product was analyzed for probiotic survivability during storage of 8 weeks using plate count assay according to the procedure of Otieno, Ashton, and Shah (2007).

Lyophilized commercially available ice cream (CAIC) or SOFY was grinded by a universal pulverizer, mixed at different proportions powder with a standard diet, and then mixed evenly using small amount of water. Finally, the mix was put into molds, stored in well‐ventilated place for use. Standard diets were purchased from Experimental Animal Center, Jilin University, and were in conformity with Chinese standard GB 14924‐2001.

### 2.4 Animals, diets, and experimental design

Eighty male Wistar rats weighing 250 ± 10 g were obtained from the Laboratory Animal Center of Jilin University. All animal experiments were conducted in accordance with the Directive 2010/63/EU and approved by the Institutional Animal Care and Use Committee of Jilin University, IACUC (Approve number: 20140405). All rats were housed in specific pathogen‐free polypropylene cages (545 × 395 × 200 mm) with stainless steel covers. The cages contained dust‐free sterilized softwood shaving as bedding materials. The animals were kept in a room maintained at 22 ± 2°C, relative humidity of 50%‐60%, and ventilation frequency of ≥ 10 times/hr, and 12‐hr light/dark cycle (light cycle from 7 a.m. to 7 p.m.) with free access to food and drinking water. All rats were conditioned for 1 week prior to the experiment, in which there were no apparent changes in general status, including body weight, food intake, skin, excretions, gait, and behavior.

The experiments were designed based on the published methods (Hsu & Huang, 2006; Chen, Liu, Zhu, Xu, & Li, 2010) and Chinese Technical Standards for Testing and Assessment of Health Food (2003). Upon completion of the adaptation period, all rats were fed with high cholesterol diet (80.60%, w/w, of standard diet, 10% lard oil, 5% sucrose, 2% yolk powder, 2% cholesterol, 0.30% sodium deoxycholate, 0.10% propylthiouracil) for 8 weeks. After that, rats with high plasma cholesterol were weighed and fifty of them were selected for further study based on baseline body weight.

The fifty rats were randomly divided into 5 groups of 10 animals each, including blank group (D), control group (C), and three test groups: low‐dose group (L), medium‐dose group (M), and high‐dose group (H). Rats in three test groups were fed with diets containing 25% (L), 30% (M), and 35% (H) of the oat‐based frozen yogurt, respectively, compared with the rats fed with a diet containing 30% commercial ice cream (C), and a blank group fed with a standard diet (D) for another 8 weeks.

### 2.5 Body weight and related organ weight measurement

Body weights of all rats were measured each week during 16 weeks. Relative weight of each organ (or paired organs) was calculated based on final individual body weight measured on the day of termination.

### 2.6 Serum lipids profile analysis

At the end of the experiment (after 16 weeks), all rats were sacrificed after fasting overnight. Blood samples were collected from the abdominal aorta using heparin anticoagulant under diethyl ether anesthesia. Plasma was obtained by centrifugation at 1089 g for 10 min at 4°C and stored at −80°C until analysis. Immediately after blood collection, rats were killed by decapitation and livers were then removed and stored at −80°C until use.

Serum triacylglycerols (TG), total cholesterol (TC), high‐density lipoprotein cholesterol (HDL‐c), and low‐density lipoprotein cholesterol (LDL‐c) levels were measured by enzymatic and colorimetric methods using a HITACHI 7060 automatic biochemical analyzer (7060, Hitachi Co., Ltd, Tokyo, Japan).

### 2.7 Liver histological analyses

The liver histology was analyzed by the method of Castro et al., 2013 Fragments of liver were fixed in 10% (v/v) formalin. After 72 hr of fixation, the fragments were dehydrated, cleared, and embedded in paraffin. Paraffin blocks were cut into 4‐μm‐thick sections and stained by hematoxylin and eosin for assessment of architectural damage and the inflammatory process by optical microscopy.

### 2.8 Statistical analyses

All analyses were performed in triplicates. All statistical analyses were performed using the statistical program SPSS Version 11.5. (SPSS Inc. Chicago, IL, USA). ANOVA was applied to analyze the differences between groups of data. Results were expressed as mean ± standard deviation (*SD*). Statistical significance was set at α = 0.05. All figures were made using Origin Pro v8.0 (Origin Lab Ltd., Northampton, USA).

## 3 RESULTS AND DISCUSSION

### 3.1 Chemical composition and probiotic survivability analyses

**Figure 2:**
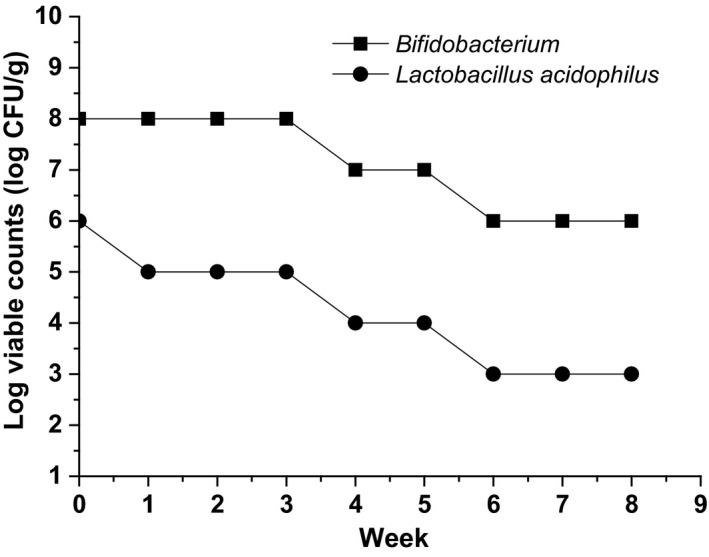
Survivability of *Bifidobacterium* and *Lactobacillus acidophilus* during storage (−18°C). Error bars are ±*SD* of the means (*n* = 10 for each group)

The SOFY was developed as a new oat‐based functional ice cream like product, which could be consumed by the patients with CVD. The formula was determined in our preliminary study where influences of main ingredients on the quality of ice cream including overrun, melt rate, hardness, viscosity, and sensory properties were studied. Chemical composition of the product is shown in Table 1. Total solids of the new product were 34.30 ± 0.19%, and protein, fat, and fiber were 4.10 ± 0.09, 8.50 ± 0.15, and 2.10 ± 0.02%, respectively. The new product was also evaluated for sensory attributes including appearance, body and texture, flavor, and taste, and the overall score is 91.83 ± 1.2. The probiotic survivability of SOFY during 8 weeks of storage at −18°C is shown in Figure 2. The population of viable *Bifidobacterium* and *Lactobacillus acidophilus* decreased during storage. However, *Bifidobacterium* still remained high number of viable cells. The initial population of *Bifidobacterium* in the experimental frozen yogurt 7.56 × 10^8 ^CFU/ml decreased to 8.40 × 10^6^ CFU/ml. Similarly, the initial population of *Lactobacillus acidophilus* in the experimental frozen yogurt was 6.32 × 10^6^ CFU/ml and down to 5.60 × 10^3 ^CFU/ml. This result indicated that *Bifidobacterium* survived much better during 8‐week storage at −18°C compared with *Lactobacillus acidophilus*.

**Table 1:** Chemical composition of symbiotic oat‐based frozen yogurt (SOFY)

Total solids (%)	34.30 ± 0.19	Iron (mg/100 g)	2.28 ± 1.30
Protein (%)	4.10 ± 0.09	Calcium (mg/100 g)	275.31 ± 23.81
Fat (%)	8.50 ± 0.15	Zinc (mg/100 g)	0.64 ± 0.03
Carbohydrate (%)	22.00 ± 0.20		
Ash (%)	0.30 ± 0.00		
Fiber (%)	2.10 ± 0.02		

### 3.2 Effects of SOFY on body weight and related organ weight in high cholesterol rats

**Figure 3:**
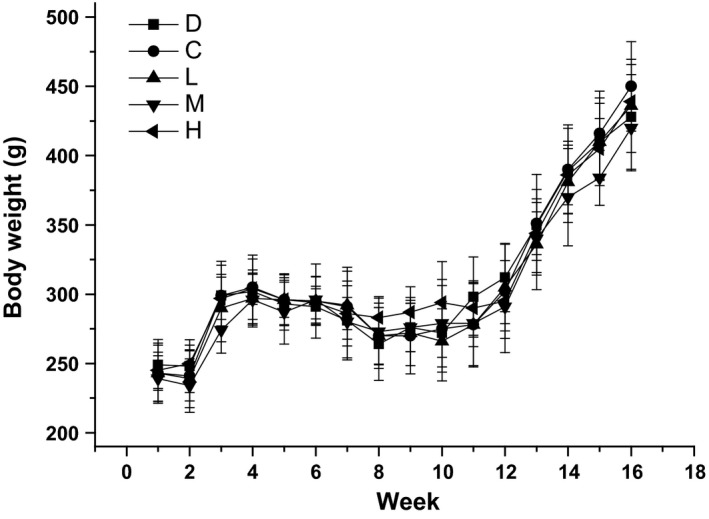
Effects of symbiotic oat‐based frozen yogurt (SOFY) on body weight of rats fed with high cholesterol diet. Error bars are ±*SD* of the means (*n* = 10 for each group)

Hyperlipidemia was associated with changes in body weight and liver, kidney and spleen weight compared to the normal control rats. Therefore, we recorded the body weights and the three organ weights for all the rats. Effects of SOFY on the body weight in rats of hyperlipidemia are shown in Figure 3. There was no significant difference in body weight between groups of all hyperlipidemia rats over the 16 weeks. For all groups, body weight increased gradually as experiment progressed in the first 4 weeks and then downwarded in the conditioning period. And then body weight of hyperlipidemia rats increased gradually during the following treatment period. Hyperlipidemia rats fed on SOFY gained lower body weight than those of on standard and commercial samples suggesting that SOFY could prevent excessive weight gain. Effects of SOFY on weight of related organs of rat models of hyperlipidemia are shown in Table 2. For liver weight, significant differences between H groups and M groups were observed (*p* < 0.05). Lowest relative liver weight for the high SOFY dose group indicated that diet containing SOFY up to 35% minimized the liver damages caused by the high cholesterol diet. In addition, there was a significant difference in spleen weight between D and L, M, H groups (*p* < 0.05).

**Table 2:** Effect of symbiotic oat‐based frozen yogurt (SOFY) on the weight of related organs of rats fed with high cholesterol diet

Group	Final body weight (g)	Liver (mg/g)	Kidney (mg/g)	Spleen (mg/g)
D	428.13 ± 40.46	30.10 ± 0.31	0.65 ± 0.08	0.39 ± 0.06
C	450.11 ± 60.65	30.80 ± 0.16	0.63 ± 0.07	0.45 ± 0.07
L	436.00 ± 48.75	31.00 ± 0.17	0.62 ± 0.04	0.5 ± 0.04
M	420.29 ± 55.99	31.90 ± 0.15	0.66 ± 0.05	0.52 ± 0.04
H	439.22 ± 20.70	29.60 ± 0.36	0.62 ± 0.06	0.43 ± 0.05
Significant level		#, H‐M		#, D‐L, M, H, C‐ L, M

Values are present as mean ± *SD*,* n* = 10. # is the significant level of *p* < 0.05.

### 3.3 Effects of SOFY on blood lipid levels in high cholesterol rats

**Figure 4:**
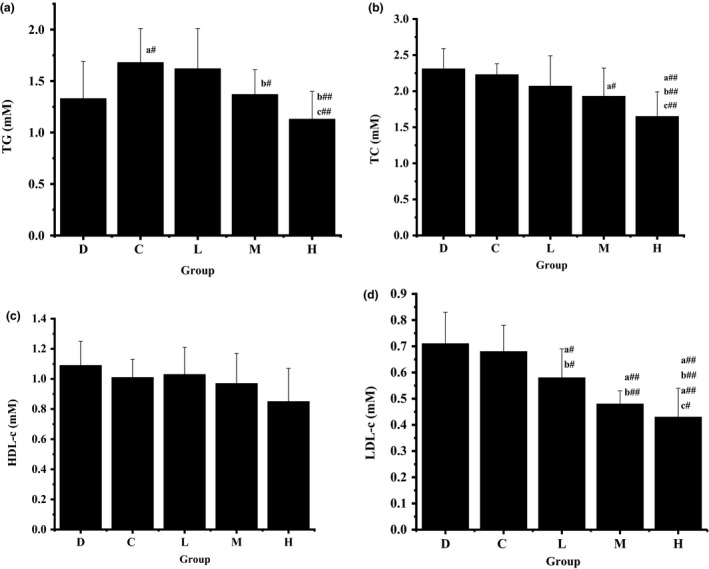
Effects of symbiotic oat‐based frozen yogurt (SOFY) on the plasma triacylglycerols (TG), total cholesterol (TC), high‐density lipoprotein cholesterol (HDL‐c), and low‐density lipoprotein cholesterol (LDL‐c) levels (mmol/L) in rats fed high cholesterol diet. The blank group (D), control group (C), low‐dose group (L), medium‐dose group (M), and high‐dose group (H) are presented. Error bars are ±*SD* of the means (*n* = 10 for each group). a, significantly different from group D; b, significantly different from group C; c, significantly different from group L. #, *p* < 0.05; ##, *p* < 0.01

Triacylglycerols is a major component of chylomicron and energy substrates for liver and peripheral tissue, particularly, muscles. However, high level of TG is a risk factor for atherosclerosis (Jain, Kathiravan, Somani, & Shishoo, 2007). Temme, Van Hoydonck, Schouten, and Kesteloot (2002) also pointed out that high level of TC is one of the predictors of atherosclerosis. Effect of SOFY on TG level in rat models of hyperlipidemia is shown in Figure 4A. The specific TG levels of groups D, C, L, M, and H were 1.33 ± 0.36, 1.68 ± 0.33, 1.62 ± 0.39, 1.37 ± 0.24, and 1.13 ± 0.27 mmol/L, respectively. At the end of the study, keeping the rats on the diet containing 30% CAIC significantly increased the TG level in serum of control group (C) as compared to the blank group (D) (*p* < 0.05). Simultaneously, the TG levels in serum of groups M and H, where the rats were fed with a diet containing 30% and 35% SOFY, respectively, significantly decreased as compared to the control group (C) (*p* < 0.05/*p* < 0.01). TG levels in serum of three experimental groups showed a decreased trend as the SOFY level increased. It is noteworthy that the TG level in serum of group H was lower than that of group D and also significantly lower than that of group L.

The effect of SOFY on the TC level in rat models of hyperlipidemia is shown in Figure 4B. The TC levels of groups D, C, L, M, and H were 2.31 ± 0.28, 2.23 ± 0.15, 2.07 ± 0.42, 1.93 ± 0.39, and 1.65 ± 0.34 mmol/L, respectively. The result displayed a clear trend that all TC levels in serum of three test groups not only were lower than that of blank group (D) or control group (C), but also decreased with the SOFY dose increased. There was no difference between group D and group C, where the rats were fed with a standard diet or a diet containing 30% CAIC, respectively. However, TC level of group M was significantly lower than that of group D (*p* < 0.05). Feeding the rats with a diet containing 35% SOFY significantly decreased the TC level in serum of high‐dose group (H) as compared to the groups D, C, and L (*p* < 0.01).

HDL‐c exerts an anti‐atherogenic effect by counteracting LDL‐c oxidation and facilitating the translocation of cholesterol from peripheral tissue such as arterial walls to the liver for catabolism (Choi, Yokozawa, & Oura, 1991). The study showed that an increase in HDL‐c is associated with a decrease in coronary risk (Miller, 1978). After 16 weeks of experimentation, effect of SOFY on the HDL‐c level in rat models of hyperlipidemia is shown in Figure 4C. The specific HDL‐c levels of groups D, C, L, M, and H were 1.09 ± 0.16, 1.01 ± 0.12, 1.03 ± 0.18, 0.97 ± 0.20, and 0.85 ± 0.22 mmol/L, respectively. Rats of control group (C) fed with a diet containing 30% CAIC had lower HDL‐c level than those of blank group (D) fed with a standard diet. Rats of low‐dose group (L) fed a diet containing 25% SOFY had higher HDL‐c level than rats of group C. Unexpectedly, the HDL‐c levels of medium‐dose group (M) and high‐dose group (H) were lower than that of group C. The HDL‐c levels in the three test groups did not show an increasing trend. However, there was no significant difference between each two groups (*p* > 0.05).

According to many studies, LDL‐c is the most important factor among serum lipids. For example, LDL‐c is known to accumulate in the extracellular subendothelial space of arteries and to be toxic to arterial walls (Rota, Mcwilliam, Baglin, & Byrne, 1998). What's more, LDL‐c level remains the primary lipid target and risk factor for atherosclerosis. The LDL‐c level is usually used as the basis for initiating and monitoring the treatment of patients with elevated blood cholesterol levels (Schaefer, Lichtenstein, Lamon‐Fava, Mcnamara, & Ordovas, 1995). The effects of SOFY on the LDL‐c level (mmol/L) in rat models of hyperlipidemia are shown in Figure 4D. The specific LDL‐c levels of groups D, C, L, M, and H were 0.71 ± 0.12, 0.68 ± 0.1, 0.58 ± 0.11, 0.48 ± 0.05, and 0.43 ± 0.11 mmol/L, respectively. The result of LDL‐c level was similar with that of TC level. All LDL‐c levels in serum of three test groups not only were lower than that of blank group (D) or control group (C), but also decreased with the SOFY dose increased. There was slight difference between group D and group C, where the rats were fed a standard diet or a diet containing 30% CAIC, respectively. However, LDL‐c levels in serum of group L (*p* < 0.05), group M (*p* < 0.01), and group H (*p* < 0.01) were significantly lower than those of group D or group C. Besides, the LDL‐c level of group H decreased significantly as compared to that of group L. The results were similar with the previous study which showed that oat milk decreased LDL‐c values (Önning, Åkesson, Öste, & Lundquist, 1998).

A.I., which is the ratio of LDL‐c to HDL‐c, is commonly used as an index to evaluate the risk of atherosclerosis as a result of increased HDL‐c levels. Therefore, increasing the level of HDL‐c and lowering the A.I. are important measures in reducing the risk of atherosclerosis. Rats fed with a diet containing SOFY (≥25%) did not show an increasing trend of HDL‐c levels, but showed a little change compared to control group. However, the administration of a diet containing SOFY (≥25%) resulted in a decreased A.I. value, with marked decreasing LDL‐c levels and no observed changes in the HDL‐c levels. Taken together, these results demonstrated that SOFY might exert hypolipidemic effects by lowering the serum lipid levels, which was beneficial to hyperlipidemic atherosclerosis and CVD to some extent.

### 3.4 Histopathological observation of the liver tissue of the rats

**Figure 5:**
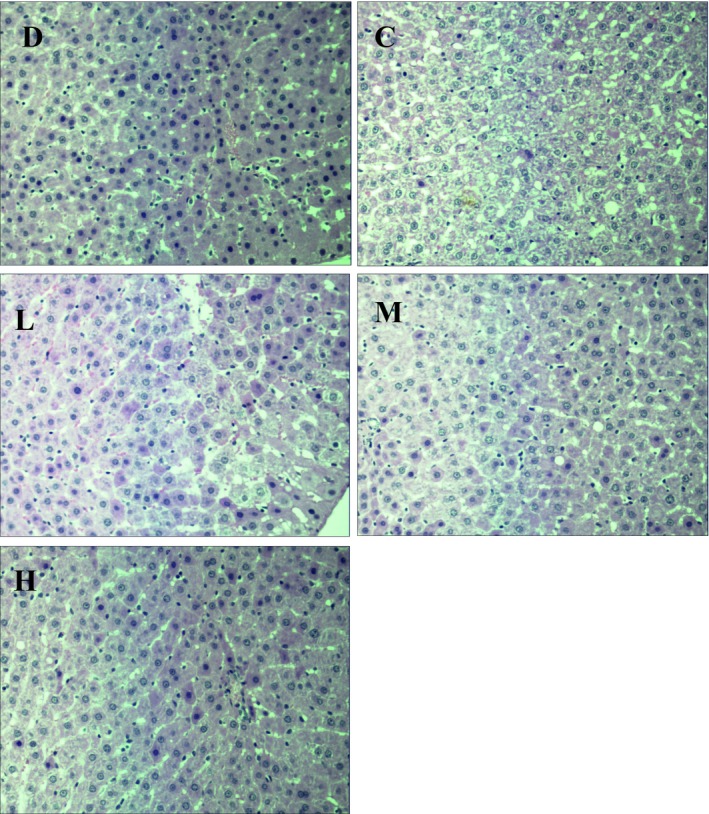
Effects of SOFY on livers of high cholesterol rats

Liver is the vital organ of metabolism, detoxification, storage and excretion of xenobiotics and their metabolites. Based on the results, the histopathological analysis was further conducted to verify the hypolipidemic effect of SOFY. Microscopic examination of liver sections is shown in Figure 5. Relatively speaking, each hepatocyte had an abundant cytoplasm, distinct cell borders, and a round, central nucleus in the blank group (D). The hepatic cell cords arranged in orderly manner, and hepatocyte morphology was normal. However, it is normal that there were little round fat vacuoles and loose, transparent cytoplasm in hyperlipidemia rats’ livers cells. In contrast, a wide spectrum of variability was found in the control group (C). Large number of liver cells had undergone degeneration, and the hepatic cords in liver were not arranged in an orderly manner. Fatty cyst and different sizes of fat droplets were observed in the cytoplasm of hepatocytes. In some cells, lipid storage accumulates in large macrovesicular globules, displacing the nucleus to the periphery of the cell.

There were positive changes in all three test groups, low‐dose group (L), medium‐dose group (M), and high‐dose group (H). As shown in Figure 5 (L, M, and H), the hepatic architecture got better with the SOFY increase. The circular fat droplets in the cytoplasm were significantly decreased, and the degeneration of the hepatocyte was markedly decreased. In addition, the liver cell structure of group L and M was more and more similar to the liver cell structure of group D. It is noteworthy that no evidence of lipid accumulation was found in the group H, and the cytoplasm was more compact in group H than that of group D. The results of this study were consistent with the analysis of blood lipids profile. All results showed a positive effect of SOFY on blood lipids in hyperlipidemia rats and a greater antihyperlipidemic effect of SOFY than that of CAIC.

In the present study, all TC and LDL‐c levels in the three experimental groups showed a marked decrease compared with those in blank (D) and control (C) groups. Although the TG levels in low‐dose group (L) and medium‐dose group (M) were slightly higher than those in group D, the TG level in high‐dose group was significantly lower than that in group D. That is to say, administration of a diet containing SOFY (≥ 25%) to hyperlipidemia rats for 8 weeks significantly reduced the levels of TC, TG, and LDL‐c in plasma. While there is no significant difference between blank group (D) and control group (C) in TC, TG, and LDL‐c levels, the results indicated that SOFY could exert hypolipidemic effects by reducing the serum TC, TG, and LDL‐c levels. Another study revealed that a hypocaloric diet containing oats consumed over 6 weeks resulted in greater improvements in lipid profile than a hypocaloric diet without oats did (Saltzman et al., 2001). Probiotics and soy protein were also reported to be effective in the management of hyperlipidemia (Stancu, Sanda, Deleanu, & Sima, 2014; Chen, Wang, Zhang, & Yang, 2014). Therefore, based on the composition, the major components responsible for hypolipidemic effect may be oat, probiotics, and soy protein in this product.

## 4 CONCLUSIONS

In conclusion, consumption of the symbiotic oat‐based frozen yogurt may decrease the levels of serum TG, total and LDL cholesterol in rats. However, this study has some limitations due to the small sample size and the single species may have differences when compared with naturally occurring diseases. In future studies, it is necessary to use rabbits or other animal experiments for comprehensive studying the effects of SOFY on the regulation of blood lipid. On the basis of animal experiments, it would be significant to test the effect on volunteer peoples.

## CONFLICT OF INTEREST

The authors declare no conflict of interest.

## ETHICAL STATEMENT

The authors declare that there is no conflict of interest regarding the publication of this article. All animal experiments were conducted in accordance with the Directive 2010/63/EU and approved by the Institutional Animal Care and Use Committee of Jilin University, IACUC (Approve number: 20140405).
